# The efficacy of non-depolarizing muscle relaxant as adjuvants to local anesthesia in peribulbar block during cataract surgery

**DOI:** 10.1038/s41598-025-87042-3

**Published:** 2025-01-29

**Authors:** Ainelmarwa Abdelmonem Abdallah Hassan, Fatma Alzahraa Roshdy Elkemary, Heba Mahmoud Abdelrahman

**Affiliations:** 1 Department of Anesthesia, Intensive Care & Pain Management, Faculty of Medicine for Girls, Al-Azhar University, Cairo, Egypt; 2 Department of Ophthalmology, Faculty of Medicine for Girls, Al-Azhar University, Cairo, Egypt

**Keywords:** Peribulbar anesthesia, Rocuronium, Atracurium, Health care, Health occupations, Medical research

## Abstract

Peribulbar anesthesia is mainly used for cataract surgery. Many studies had used atracurium and rocuronium as an additive to the local anesthetic (LA) drugs in eye surgery. The aim of this study is to evaluate the efficacy of adding atracurium versus rocuronium to a local anesthetic mixture, in providing an early onset of orbital akinesia and corneal anesthesia during cataract surgery. Ninety-three patients met the inclusion criteria and gave a written informed consent to participate. Patients were randomly allocated in three study groups (31 patients in each group). Group I received either 3 mL of a lidocaine2%(mixed by hyaluronidase 50 IU/ml) and 3 ml bupivacaine mixture, plus 0.5 mL rocuronium (5 mg) total volume 6.5 ml. Group II 3 mL of a lidocaine2%(mixed by hyaluronidase 50 IU/ml) and 3 ml bupivacaine mixture plus 0.5 mL (5 mg) atracurium total volume 6.5 ml. Group III or3 mL of a lidocaine2%(mixed by hyaluronidase 50 IU/ml) and − 3 ml bupivacaine mixture, plus 0.5 mL saline total volume 6.5 ml. The onset time of orbital akinesia was significantly lower in the rocuronium group and the atracurium group compared to the control group. Akinesia score was (0.90 ± 0.30) and (0.19 ± 0.40) in rocuronium group at 4 min and 6 min respectively which result in highly significant difference than atracurium and control groups. The use of rocuronium and atracurium will result in rapid orbital akinesia and rapid orbicularis muscle akinesia.

## Introduction

Local anesthesia of the orbit during cataract surgery has been used over many decades. Surgeons have been using local anesthesia since 1884 for ophthalmic procedures^1^. Local anesthesia is much safer than general anesthesia, as most of the patients are geriatrics, which means more morbidities and mortalities could happen during the surgery^2^. So many anesthetic techniques have been developed and many anesthetic drugs have been used, to bring the optimal and save surgical condition. Today, local anesthesia is being used in cataract surgery in the form of retro bulbar, peribulbar blocks or topical anesthesia. The main task of anesthesia during cataract surgery is akinesia and corneal anesthesia, which helps the surgeon during the operation^3^.

Peribulbar anesthesia is mainly used for cataract surgery. This technique is associated with fewer serious complications compared with retrobulbar anesthesia. However, it has the disadvantage of a slow onset of orbital akinesia and the frequent need for block supplementation^4^. To overcome these limitations, many adjuvant drugs have been used to fast onset and to prolong duration of local anesthetic drugs. For example, adrenalin, hyaluronic acid, bicarbonate, opioid and ketamine^5^. Many studies had used neuromuscular blocking drugs (NMBD), namely atracurium and rocuronium as an additive to the local anesthetic (LA) drugs in eye surgery. These studies had found that the use of NMBD have fasten the onset and prolong the duration of LA^6,7^.

Atracurium and rocuronium act through competitive antagonizing neurotransmitter action of acetylcholine at the cholinergic receptor sites on the motor endplate. Atracurium is a relatively new NMBD which has a unique mode of elimination, by spontaneous degradation (Hofmann degradation). Atracurium has an onset of action of approximately 2 min when an intubating dose is given (0.4–0.6 mg). The side effect of atracurium includes, skin flush, itching, wheezing and allergic reaction. And these effect have limited it is use in atopic patient and patient with history of bronchospasm^8^.

The onset of action of rocuronium is different from that of atracurium as it takes only from 60 to 90 s for optimal tracheal intubation condition. The intubating dose is 0.6 to 1.2 mg/kg and last for approximately 45 min. Bronchospasm and anaphylaxis are recognized complication of rocuronium^9^.

Despite the use of atracurium and rocuronium in cataract surgery, no study compared the atracurium versus rocuronium. Thus the aim of this study is to evaluate the efficacy of adding two different non depolarizing muscle relaxant drugs (namely atracurium and rocuronium) to a local anesthetic mixture in providing an early onset of orbital akinesia and corneal anesthesia during cataract surgery.

## Patients and methods

We confirm that all methods were carried out in accordance with relevant guidelines and regulations of Alzahra hospital (Alazhar university) after receiving approval from the ethics committee of Alzahra hospital (Alazhar university, AFMG-IRB 1947), and informed consent was obtained from all patients included in this study.100 adult patients of both sexes, with ASA grade I and II, in the age group 40–70 years, scheduled for elective cataract surgery under local anesthesia, were admitted to the ophthalmology operating theater and enrolled in a prospective randomized controlled trial. Exclusion Criteria from the study included; patient refusal, patients with history of allergy to local anesthetic drugs, history of chronic pain, drug addiction, complicated surgery. However only ninety-three meet the inclusion criteria and gave a written informed consent to participate.

The patients were randomly allocated in three study groups (31 patients in each group). Group I received either 3 mL of a lidocaine2%(mixed by hyaluronidase 50 IU/ml) and 3 ml bupivacaine mixture, plus 0.5 mL rocuronium (5 mg) total volume 6.5 ml. Group II 3 mL of a lidocaine2%(mixed by hyaluronidase 50 IU/ml) and 3 ml bupivacaine mixture plus 0.5 mL (5 mg) atracurium total volume 6.5 ml. Group III or3 mL of a lidocaine2%(mixed by hyaluronidase 50 IU/ml) and − 3 ml bupivacaine mixture, plus 0.5 mL saline total volume 6.5 ml.

If adequate condition to begin surgery was not obtained 10 min after performing the block, supplemental injection with 1–2 ml of lidocaine2%(mixed by hyaluronidase 50 IU/ml) and1ml bupivacaine mixture.

Sample size calculation was based on ocular movement score between group with atracurium, rocuronium versus control group retrieved from previous research by Mostafa et al.^10^. Using G power program version 3.1.9.4 to calculate sample size based on expected difference of 35%, using 2-tailed test, α error = 0.05 and power = 80.0%, the total calculated sample size will be 31 in each group at least.

The primary and secondary outcome were recorded by a single observer.

Primary outcome was obtained by Assessing the orbital akinesia. The patients were asked to look lateral, medial, superior, and inferior. Orbital akinesia was assessed on a 0–2 score (0 = no movement,1 = sluggish ,2 = normal) at 2 min ,4 min, 6 min and 8 min intervals. The onset of complete akinesia was recorded.

Secondary outcome was obtained by recording orbicularis muscle akinesia. The patient was then asked to forcefully close his/her eyes at time interval of 2 min, 4 min, 6 min and 8 min, to assess the orbicularis muscle on a scale of 0–2 (0 = complete akinesia, 1 = partial movement, and 2 = pronounced movement). Requirement of supplemental and undesirable local side effect were also recorded.

### Statistical analysis

Recorded data were analyzed using the statistical package for social sciences, version 23.0 (SPSS Inc., Chicago, Illinois, USA). The quantitative data were presented as mean ± standard deviation and ranges when their distribution was parametric (normal) while non-normally distributed variables (non-parametric data) were presented as median with inter-quartile range (IQR). Also qualitative variables were presented as number and percentages. Data were explored for normality using Kolmogorov-Smirnov and Shapiro-Wilk Test. The following tests were done, A one-way analysis of variance (ANOVA), Post Hoc test: Tukey’s test, Kruskall Wallis test, Mann Whitney U test, Chi-square test and Fisher’s exact test^10,11^.

## Results

Patients’ characteristics were comparable among the three groups, as well as the duration of surgery, and volume of injectate. It was found that there were no significant differences between the study groups as regards the age, sex, wight, ASA physical status distribution, duration of surgery and volume of injectate with a *p*-value > 0.05 (Table 1).

**Table 1:** Comparison between groups according to Demographic data.

Demographic Data	Atracurium group (*n* = 31)	Rocuroinum group (*n* = 31)	Control group (*n* = 31)	Test value	*p*-value	Sig.
Sex						
Female	16 (51.6%*)*	14 (45.2%*)*	12 (38.7%*)*	1.042	0.594	NS
Male	15 (48.4%*)*	17 (54.8%*)*	19 (61.3%*)*			
Age (years)						
Mean ± SD	55.52 ± 8.65	57.58 ± 8.23	58.68 ± 7.91	1.168	0.316	NS
Range	42–70	44–70	42–68			
Wt. (kg)						
Mean ± SD	87.90 ± 9.20	91.29 ± 7.63	90.00 ± 6.71	1.446	0.241	NS
Range	70–100	80–100	80–100			
Duration of surgery (min)						
Mean ± SD	36.87 ± 5.78	37.90 ± 5.44	39.68 ± 4.99	2.132	0.125	NS
Range	30–45	30–45	30–45			
ASA						
I	12 (38.7%*)*	15 (48.4%*)*	7 (22.6%*)*	7.755	0.101	NS
II	19 (61.3%*)*	16 (51.6%*)*	24 (77.4%*)*			

Using: One way Analysis of Variance test was performed for Mean ± SD.

x2: Chi-square test for Number (%) or Fisher’s exact test, when appropriate.

NS: Non significant.

The onset time of orbital akinesia was significantly lower in the rocuronium group and the atracurium group compared to the control group, and in the rocuronium compared to the atracurium group (*P* < 0.001) (Table 2).

**Table 2:** Comparison between groups according to Onset of orbital akinesia (min).

Onset of akinesia (min)	Atracurium group (*n* = 31)	Rocuroinum group (*n* = 31)	Control group (*n* = 31)	Test value	*p*-value	Sig.
Mean ± SD	5.19 ± 0.54B	4.00 ± 0.45 C	6.56 ± 0.59 A	182.255	0.001	HS
Range	4–6	3–5	6–8			

Using: One way Analysis of Variance test was performed for Mean ± SD & Multiple comparison between groups through Post Hoc test: Tukey’s test.

Different capital letters indicate significant difference at (*p* < 0.05) among means in the same row.

HS: Highly significant.

Akinesia score was (0.90 ± 0.30) and (0.19 ± 0.40) in rocuronium group at 4 min and 6 min respectively which result in highly significant difference than atracurium and control groups. At 8 min there was no significant difference between rocuronium and atracurium groups (0.00 ± 0.00) but it was highly significant in control group (0.39 ± 0.50) (Table 3).

**Table 3:** Comparison between groups according to Akinesia score.

Akinesia score	Atracurium group (*n* = 31)	Rocuroinum group (*n* = 31)	Control group (*n* = 31)	Test value	*p*-value	Sig.
Akinesia score at 2 min						
Mean ± SD	2.00 ± 0.00	2.00 ± 0.00	2.00 ± 0.00	0.000	1.000	NS
Median (IQR)	2 (2–2)	2 (2–2)	2 (2–2)			
Range	2–2	2–2	2–2			
Akinesia score at 4 min						
Mean ± SD	1.61 ± 0.50	0.90 ± 0.30	2.00 ± 0.00	85.769	0.000	HS
Median (IQR)	2 (1–2)A	1 (1–1)B	2 (2–2)A			
Range	1–2	0–1	2–2			
Akinesia score at 6 min						
Mean ± SD	0.55 ± 0.51	0.19 ± 0.40	1.23 ± 0.43	42.788	0.000	HS
Median (IQR)	1 (0–1)A	0 (0–0)B	1 (1–1)A			
Range	0–1	0–1	1–2			
Akinesia score at 8 min						
Mean ± SD	0.00 ± 0.00	0.00 ± 0.00	0.39 ± 0.50	18.947	0.000	HS
Median (IQR)	0 (0–0)B	0 (0–0)B	0 (0–1)A			
Range	0–0	0–0	0–1			

IQR: Interquartile range.

Using: Kruskal–Wallis was performed for Median (IQR) & Multiple comparison between groups through Mann-Whitney test.

Different capital letters indicate significant difference at (*p* < 0.05) among means in the same row.

*p*-value > 0.05 is insignificant; *p-value < 0.05 is significant; **p-value < 0.001 is highly significant.

However, the onset of akinesia was statistically significant but it was not clinically significant.

Orbicularis muscle akinesia score was (1.16 ± 0.37) and (0.00 ± 0.00) in rocuronium group at 4 min and 6 min respectively which result in highly significant difference than atracurium and control groups. At 8 min there was no significant difference between rocuronium and atracurium groups but it was highly significant in control group (*p* = 0.000) (Table 4).

**Table 4:** Comparison between groups according to Orbicularis muscle akinesia at 2 min, 4 min, 6 min and at 8 min.

Orbicularis akinesia	Atracurium group (*n* = 31)	Rocuroinum group (*n* = 31)	Control group (*n* = 31)	Test value	*p*-value	Sig.
Orbicularis akinesia at 2 min						
Mean ± SD	2.00 ± 0.00	2.00 ± 0.00	2.00 ± 0.00	0.000	1.000	NS
Median (IQR)	2 (2–2)	2 (2–2)	2 (2–2)			
Range	2–2	2–2	2–2			
Orbicularis akinesia at 4 min						
Mean ± SD	1.61 ± 0.50	1.16 ± 0.37	1.74 ± 0.44	14.834	0.000	HS
Median (IQR)	2 (1–2)A	1 (1–1)B	2 (1–2)A			
Range	1–2	1–2	1–2			
Orbicularis akinesia at 6 min						
Mean ± SD	1.00 ± 0.00	0.00 ± 0.00	1.00 ± 0.00	3.682	0.021	S
Median (IQR)	1 (1–1)A	0 (0–0)B	1 (1–1)A			
Range	1–1	0–0	1–1			
Orbicularis akinesia at 8 min						
Mean ± SD	0.13 ± 0.34	0.00 ± 0.00	0.48 ± 0.51	15.603	0.000	HS
Median (IQR)	0 (0–0)B	0 (0–0)B	0 (0–1)A			
Range	0–1	0–0	0–1			

IQR: Interquartile range.

Using: Kruskal–Wallis was performed for Median (IQR) & Multiple comparison between groups through Mann-Whitney test.

Different capital letters indicate significant difference at (*p* < 0.05) among means in the same row.

*p*-value > 0.05 is insignificant; *p*p*value < 0.05 is significant; **p-value < 0.001 is highly significant.

**Fig. 1:**
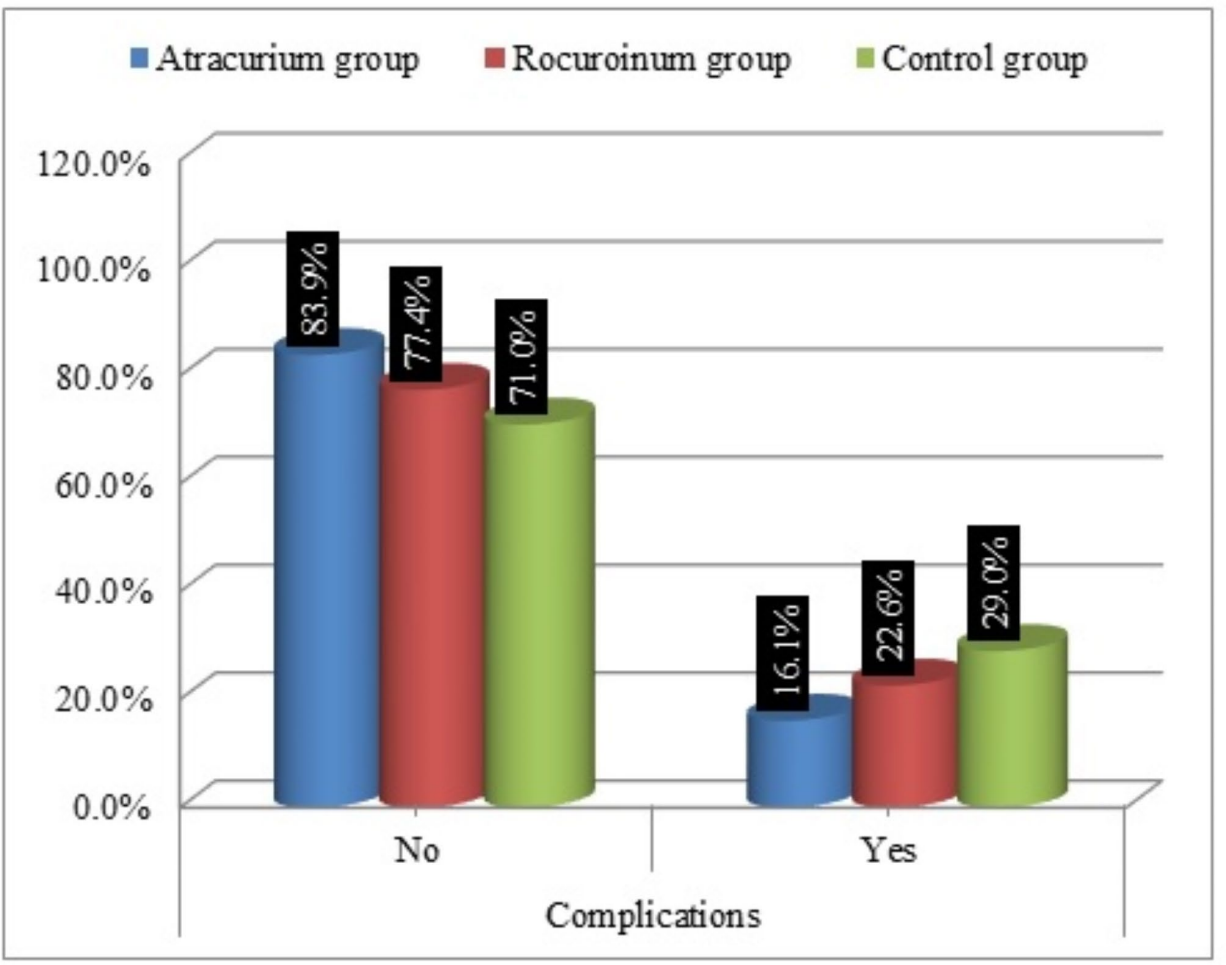
Comparison between groups according to Complications.

In atracurium group, the incidence of complication in the form of Subconjunctival edema, Subconjunctival hemorrhage, was (16.1%) which was lower compared to rocuronium group (22.6%) and controlled group (29.0%) (Fig. 1).

Further-more, fewer patients in the atracurium group required supplementation compared to rocuronium group and control group which was a statistically significant (*p* = 0.047) (Table 5).

**Table 5:** Comparison between groups according to the of incidence of supplementation requirement.

Incidence of supplementation	Atracurium group (*n* = 31)	Rocuroinum group (*n* = 31)	Control group (*n* = 31)	Test value	*p*-value	Sig.
No	28 (90.3%)	24 (77.4%)	20 (64.5%)	5.905	0.047	S
Yes	3 (9.7%) *C*	7 (22.6%) *B*	11(35.5%)*A*			

Different capital letters indicate significant difference at (*p* < 0.05) among means in the same row.

S: Significant.

## Discussion

In this randomized double blind study, we wanted to evaluate and compare the effect of non-depolarizing muscle relaxant, namely rocuronium and atracurium, as an additive to the local anesthetics during peribulbar block in cataract surgery. The study showed that the rocuronium affect the onset time of the orbital akinesia and orbital akinesia score was more than the atracurium. Also the effect of rocuronium on the orbicularis muscle akinesia was more significant than the atracurium. There were no major local or systemic complications in this study.

In a randomized controlled trial dony by Aissaoui etal^12^, Rocuronium group demonstrated significantly better akinesia scores than control group at 2, 5 and 10 min (*p* < 0.05) with no significant complications.

A study by Messeha et al.^13^, showed that Globe akinesia and akinesia score were achieved rapidly in rocuronium group than in hyaluronidase group at 2, 5 and 10 min.

In a study by Abdellatif et al.^14^, they inject a total volume of 8.5 ml of LA plus rocuronium. This result in shortened the onset time of peribulbar anesthesia and the score decreased at 2 min after block administration.In that study the very rapid onset at 2 min may resulted from tha large volum of LA compared to our study which was only 6.5 ml.

A randomized double-blinded study by Patil et al.^15^, showed the cases who received a mixture of rocuronium 5 mg with 2% lignocaine with adrenaline achieved orbital akinesia by the end of 2 min. In this study despite using 6.5 ml as in our study, the using of lidocaine with adrenaline may resulted in more rapid onset of orbital akinesia. As bupivacian may result in delayed onset than lidocaine^16,17^.

Küçükyavuz and Arici^6^, added 0.5 mL (5 mg) atracurium on 8 mL of the LA mixture and added 0.5 mL 0.9% NaCl on 8 mL of the LA mixture found that the rate of complete akinesia was not significant. But the success rate of complete akinesia was 100% with the atracurium.

Nazmy et al., adding 5 mg atracurium to LA which significantly reduced the time of onset of globe akinesia^18^.

Another study by Eghbal et al., they found that adding 5 mg (0.5) of atracurium to only 2 ml of lidocaine in retrobulber block result in rapid onset (4.7 ± 1.1 min) and prolonged duration (104.07 ± 17.6 min) of the block compared to the controlled group which was (6.9 ± 0.96 min) and (87.1 ± 16.2) respectively^19^.

Some reports showed the effect of rocuronium, atracurium, and cis-Atracurium on the local anesthetics during cataract surgery. There had been a study by Sharkawy et al., showed that adding 5 mg (0.5 ml) rocuronium versus adding 2.5 mg (0.5 ml) cia-atracurium to LA result in shorten the block onset time^20^.

Also El-Serwi et al., compared the effect of rocuronium to cia- atracurium in prebulber block, showed that The number of patients with complete akinesia was higher in the rocuronium group after than in the cia-atracurium with no statistically significant difference regarding lid akinesia^21^.

A result by Godarzi et al., found that the quality of akinesia in the 10th minute in the Atracurium group, cis-Atracurium group and the placebo group were 92.6%, 85.2% and 85.2% respectively (*P* = 0.088)^22^.

Elgohary et al., showed that there was a significant increase in time to begin surgery in control group (9.83 ± 3.34) when compared with atracurium group (5.70 ± 3.06) and cia-atracurium group (6.70 ± 2.98)^23^.

On the other hand, the complications that showed up in this study with the atracurium were lower than with the rocuronium (16.1% and 22.6%) respectively. Although these complications were statically significant, conjunctival edema and subconjunctival hemorrhage may occur after needle block. These complications are more frequent during peribulbar block than retrobulbar block, due to anterior spread of the LA and the damage of minor blood vessels with needle tip. These minor complications usually do not interfere with surgery and resolve spontaneously within few hours^24^.

Although this study demonstrated the benefits of using atracurium and rocuronium in local anesthesia during cataract surgery, there are potential limitations to their use in eye surgery. Topical betamethasone, commonly used for the management and prevention of noninfectious ocular inflammation following cataract surgery^25^, may increase the risk or severity of myopathy and weakness affecting ocular muscles when used in conjunction with atracurium or rocuronium^26^.

Additionally, gentamicin (an aminoglycoside antibiotic), often used as an ophthalmic preparation to treat eye infections, enhances the effects of atracurium and rocuronium through pharmacodynamic synergism. This interaction should be considered when using atracurium in local anesthesia for eye surgeries.

The use of atracurium besylate from multi-dose vials containing benzyl alcohol as a preservative is contraindicated in patients with a known hypersensitivity to benzyl alcohol. However, this limitation does not apply to single-dose vials of atracurium, which are preservative-free.

It is worth noting that rocuronium bromide is preservative-free, which provides an advantage over atracurium for patients with hypersensitivity or allergic reactions.

Regarding cost, rocuronium is more expensive than atracurium, which may be a limiting factor in resource-constrained settings.

Finally, caution must be exercised in the storage and handling of both atracurium and rocuronium, as their potency can be affected by temperature and light.

All previous studies work on the effect of atracurium and rocuronium in cataract surgery focused on studying the effect one of these two drugs. However, our study compared for the first time the effect atracurium versus rocuronium.

### Study limitation

In our study despite the favor result for the use of rocuronium, there was statistically significant lower incidence of additional supplementation of LA in the atracurium group, followed by the rocuronium group, then the control group, with a *p*-value (*p* = 0.032). This may result from the relatively low dose of LA used in performing the block without taking the eye globe axial length in consideration. El Fawal et al., have reported that The minimum effective volume of local anesthetics for peribulbar block show a strong and inverse correlation with eye globe axial length. This may help achieving an effective block with minimum complications^27^. Also we bypassed the assessment in post-anesthesia care unit and hence, the time taken to complete recovery orbital akinesia and the post-operative analgesia were not recorded in this study.

### Conclusion

By reviewing the overall results, we claim that the use of rocuronium and atracurium will result in rapid orbital akinesia and rapid orbicularis muscle akinesia.

## Data Availability

The datasets analyzed during the current study available from the corresponding author on reasonable request.
